# Oocyte death is triggered by the stabilization of TAp63α dimers in response to cisplatin

**DOI:** 10.1038/s41419-024-07202-7

**Published:** 2024-11-07

**Authors:** Amirhossein Abazarikia, Wonmi So, Shuo Xiao, So-Youn Kim

**Affiliations:** 1 Olson Center for Women’s Health, Department of Obstetrics and Gynecology, College of Medicine, University of Nebraska Medical Center, Omaha, NE USA; 2 Department of Pharmacology and Toxicology, Ernest Mario School of Pharmacy, Environmental and Occupational Health Sciences Institute, Rutgers Unversity, Piscataway, NJ USA; 3 Fred and Pamela Buffett Cancer Center, University of Nebraska Medical Center, Omaha, NE USA

**Keywords:** Phosphorylation, Apoptosis, Checkpoint signalling

## Abstract

The TAp63α protein is highly expressed in primordial follicle oocytes, where it typically exists in an inactive dimeric form. Upon DNA damage, TAp63α undergoes hyperphosphorylation, transitioning from a dimeric to a tetrameric structure, which initiates oocyte apoptosis by upregulating pro-apoptotic gene. Our results demonstrate that cisplatin, an alkylating anti-cancer agent, predominantly produced the TAp63α dimer rather than the tetramer. We further observed that TAp63α protein accumulation occurred in primordial follicle oocytes following cisplatin treatment, and this accumulation was significantly reduced by cycloheximide, a protein synthesis inhibitor. These findings suggest that TAp63α accumulation is driven primarily by de novo protein synthesis in response to DNA damage. Notably, cycloheximide protected oocytes from cisplatin-induced apoptosis, as evidenced by reduced levels of both PUMA, a known pro-apoptotic target gene of TAp63α, and TAp63α itself. Additionally, TAp63α turnover appears to be regulated by ubiquitination and proteasome degradation, as evidenced by TAp63α accumulation without oocyte death when treated with PYR-41, a pharmacological inhibitor. However, when TAp63α was stabilized by PYR-41 and subsequently activated by cisplatin, oocyte death occurred, marked by increased γH2AX and Cleaved PARP. Moreover, the Casein kinase 1 inhibitor PF-670462 effectively blocked cisplatin-induced oocyte death, indicating that CK1-mediated phosphorylation is essential for TAp63α activation, even in the absence of tetramer formation. The ATR inhibitor BEZ235 prevented cisplatin-induced TAp63α accumulation, suggesting that TAp63α accumulation precedes its phosphorylation-driven activation. Collectively, our study reveals a novel mechanism of cisplatin-induced apoptosis in primordial follicle oocyte through TAp63α stabilization and accumulation, independent of tetramerization.

## Introduction

Women are born with a finite supply of primary oocytes in their ovaries [1, 2]. The number of primordial follicles constitutes the ovarian reserve, which determines a woman’s reproductive lifespan. Primordial follicles are highly sensitive to genotoxic treatments [3–5]. Exposure to genotoxins can cause follicle loss, leading to primary ovarian insufficiency (POI), early menopause, infertility, and associated complications such as osteoporosis, depression, heart disease, and dementia [6, 7].

TAp63α, a member of p53 family, exhibits high expression in the oocytes of primordial follicles [8, 9] and remains primarily in the nuclei of these follicles throughout the reproductive lifespan [9, 10]. In newborn ovaries at postnatal day 0.5 (PD0.5), TAp63α is expressed at a markedly low level, making these oocytes tolerate to γ-radiation. However, by PD5, TAp63α becomes robustly expressed in nearly all primordial follicles, rendering them highly sensitive to DNA damage [11]. In response to DNA damage, TAp63α undergoes rapid phosphorylation-induced tetramerization and activation. Checkpoint kinase 2 (CHEK2) and casein kinase 1 (CK1) phosphorylate specific sites on TAp63α (S582 by CHEK2 and S585, S588, S591, and T594 by CK1), transforming its structure and leading to the formation of active tetramers [12–16]. Once TAp63α is hyperphosphorylated and tetramerization in response to DNA damage, oocytes in primordial follicles loss TAp63α protein expression and begin to express apoptotic markers such as BAX [17]. Results from a conditional knockout mouse model with oocyte-sepecific deletion of *Trp63* support the crucial roles of TAp63α in oocyte death induced by genotoxic agents [3, 17, 18]. Interestingly, TAp63α exhibits distinct activation patterns in response to different genotoxins [3].

A previous study demonstrated that TAp63α can be regulated at both the transcriptional and translational levels [19]. It was found that all TA isoforms are transiently expressed and then degraded via the proteasome pathway. Gonfloni and colleagues showed that cisplatin (CDDP) induces the accumulation of TAp63α protein in oocytes ex vivo in a cABL-dependent manner [20]. However, the results from other two studies suggest that ABL kinases are dispensable for the activation of TAp63α [3, 18]. In this study, we aimed to investigate the regulatory mechanism of TAp63α protein in response to CDDP. We elucidated that upon treatment with CDDP, TAp63α protein in primordial follicle oocytes becomes stabilized and accumulates before undergoing phosphorylation, and TAp63α induces oocyte death in its dimer form.

## Results

### TAp63α accumulates in oocytes of primordial follicles without complete tetramerization in vivo

**Fig. 1:**
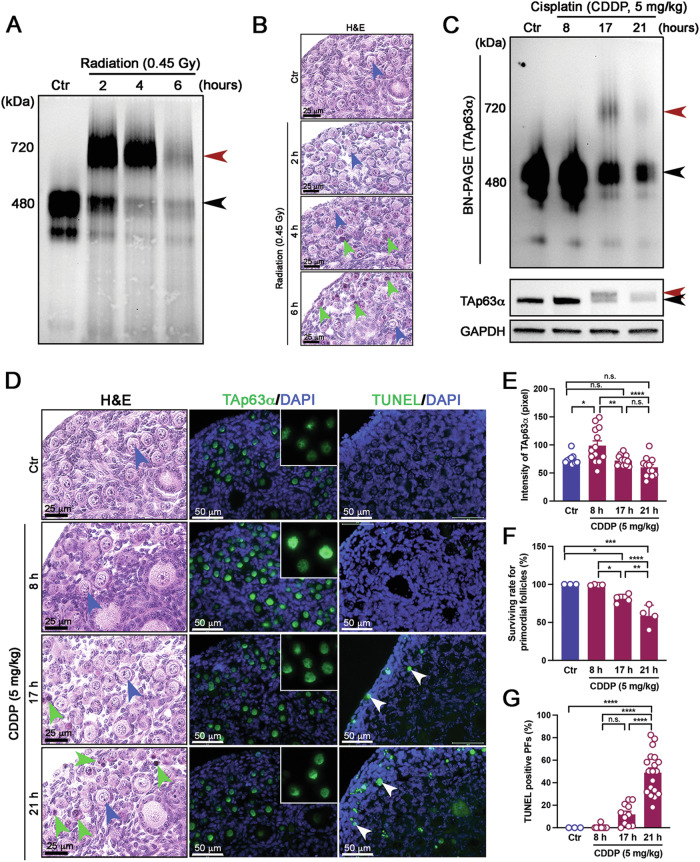
TAp63α is accumulated in dimeric forms following CDDP injection. **A** Immunoblot analysis of TAp63α on BN-PAGE using ovarian extracts from mice exposed to 0.45 Gy irradiation. Ovaries were harvested at 0, 2, 4, and 6 h post-exposure. The molecular weights of the TAp63α dimer (480 kDa, a black arrowhead) and tetramer (720 kDa, a red arrowhead) are indicated on the BN-PAGE. **B** Histological analysis with H&E staining on ovarian samples from mice exposed to 0.45 Gy irradiation. Intact primordial follicles are marked with blue arrowheads, while apoptotic follicles are marked with green arrowheads. **C** Immunoblot analysis of TAp63α on BN-PAGE, and TAp63α and GAPDH on SDS-PAGE, using ovarian extracts from mice treated with either solvent (Control, Ctr) or 5 mg/kg CDDP at 8, 17, and 21 h post-injection. Phosphorylated and unphosphorylated bands on SDS-PAGE are marked with red and black arrowheads, respectively. **D** Histological analysis with H&E staining, immunofluorescence assay for TAp63α, and TUNEL assay in ovarian samples from mice treated with either solvent (Control, Ctr) or CDDP at 8, 17, and 21 h post-injection. Intact primordial follicles are marked with blue arrowheads, and apoptotic ones with green arrowheads. Insets show TAp63α expression at each time point, with TUNEL-positive oocytes indicated by white arrowheads. **E** Quantification of TAp63α Intensity. **F** Survival rate of primordial follicles. **G** Quantification and calculation of TUNEL-positive primordial follicles in each ovarian samples. Statistical significance: n.s. not significant; *p < 0.05; **p < 0.01; ***p < 0.001; ****p < 0.0001.

Previous reports have demonstrated that TAp63α is hyperphosphorylated and forms tetramers in oocytes upon DNA damage [3, 12]. Consistent with these findings, 0.45 Gy irradiation induced strong tetramer bands of TAp63α, as indicated by a red arrowhead in BN-PAGE gel (Fig. 1A). Nuclear pyknosis in oocytes was observed 4 h post-irradiation, a time point at which the 480 kDa dimer bands appeared faint. Notably, the tetramer bands diminished as most primordial follicle oocytes reached a pyknotic status (Fig. 1A, B). In contrast, after intraperitoneally (i.p.) injection of mice with 5 mg/kg CDDP, TAp63α predominantly exhibited strong dimer bands on BN-PAGE gels (Fig. 1C). Although weak phosphorylation was detected on SDS-PAGE, the 480 kDa dimers of TAp63α were primarily observed within a 21-h period, with no significant tetramer formation. Interestingly, an increase in TAp63α levels were detected 8 h post-injection, followed by a gradual decline (Figs. 1C and S1). This transient accumulation is consistent with previous reports of TAp63α in ovary lysates exposed to CDDP ex vivo [20]. Histological and immunofluorescence assays showed that TAp63α accumulated at the 8-h mark while primordial follicles remained healthy (Fig. 1D, blue arrowheads). The highest intensity of TAp63α was observed at 8 h post-CDDP exposure (Fig. 1E). Conversely, a decrease in TAp63α levels was noted at the 17 and 21 h, coinciding with oocyte apoptosis within primordial follicles (Fig. 1D, green arrowheads). Primordial follicle survival and TUNEL-positive primordial follicle counts indicated that primordial follicle death was induced as TAp63α levels began to decline (Fig. 1F, G).

Similarly, the alkylating agent cyclophosphamide (CPA) induced TAp63α accumulation at 8 h (Fig. S2A) and weak phosphorylation by 17 h (Fig. S2A). However, dimer and tetramer bands of TAp63α at 480 and 720 kDa, respectively, were observed on BN-PAGE (Fig. S2A, black and red arrowheads). During CPA-induced accumulation of TAp63α, most primordial follicles remained intact without signs of oocyte apoptosis at 8 h, as corroborated by follicle survival and TUNEL assays (Fig. S2B–E). Furthermore, ex vivo exposure to 4-hydroperoxy cyclophosphamide (4-HC), a metabolite of CPA [21], corroborated the in vivo results (Fig. S2F). 4-HC induced the accumulation of TAp63α in oocytes of primordial follicles (Fig. S2G). These findings suggest that TAp63α accumulates in response to alkylating agents, yet this accumulation does not drive tetramerization or oocyte apoptosis in primordial follicles.

### TAp63α overexpression in cells also leads to the formation of strong dimer bands upon CDDP treatment

**Fig. 2:**
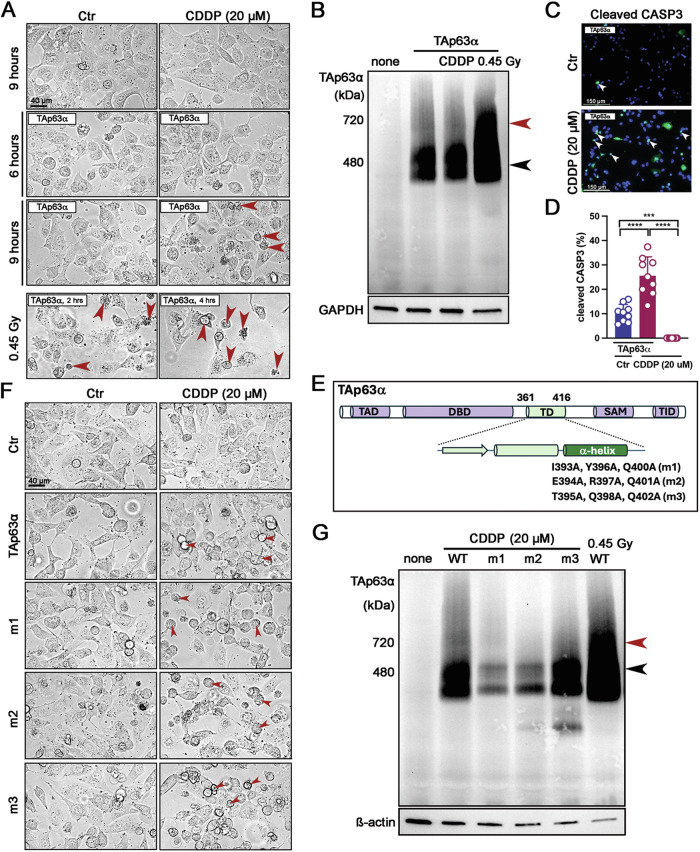
TAp63α induces apoptosis as a dimer when exposed to CDDP. **A** Images of H1299 cells treated with either solvent (Control, Ctr) or 20 µM CDDP for 9 h without TAp63α transfection, and for 6 and 9 h with TAp63α transfection. As a positive control for cell death, H1299 cells treated with 0.45 Gy irradiation were compared 2 and 4 h after TAp63α transfection. Apoptotic cells are indicated by red arrowheads. **B** Immunoblot analysis of TAp63α on BN-PAGE using cell extracts transfected with TAp63α and exposed to 20 µM CDDP for 9 h or 0.45 Gy irradiation for 2 h. The molecular weights of the TAp63α dimer (480 kDa, a black arrowhead) and tetramer (720 kDa, a red arrowhead) are indicated on the BN-PAGE. **C** Immunofluorescence assay of Cleaved CASP3 in cells treated with either solvent (Control, Ctr) or 20 µM CDDP for 9 h following TAp63α transfection. **D** Quantification of Cleaved CASP3-positive cells. **E** TAp63α plasmid constructs showing each domain and the specific mutant amino acids in the α-helix of the TD. TAD transactivation domain, DBD DNA-binding domain, TD tetramerization domain, SAM Sterile alpha motif, TID transactivation inhibitory domain. **F** Images of H1299 cells treated with either solvent (Control, Ctr) or 20 µM CDDP for 9 h, following transfection with either TAp63α or one of three TAp63α mutant plasmid constructs (m1, m2, and m3). Apoptotic cells are marked with red arrowheads. **G** Immunoblot analysis of TAp63α on BN-PAGE using cell extracts transfected with TAp63α or one of three TAp63α mutant plasmid constructs and exposed to 20 µM CDDP for 9 h. A cell extract transfected with wild-type TAp63α and exposed to 0.45 Gy irradiation for 2 h. The molecular weights of the TAp63α dimer (480 kDa, a black arrowhead) and tetramer (720 kDa, a red arrowhead) are indicated on the BN-PAGE. Statistical significance: ***p < 0.001; ****p < 0.0001.

To clarify the specificity of TAp63α dimerization upon CDDP treatment, H1299 cells were chosen for overexpression of TAp63α due to their lack of both p63 and p53 expression. H1299 cells exposed to 20 µM CDDP for 6 h without the TAp63α plasmid construct remained healthy (Fig. 2A). In contrast, cells transfected with the TAp63α plasmid construct and exposed to 20 μM CDDP exhibited apoptosis, indicated by red arrowheads, similar to cells exposed to 0.45 Gy irradiation for 2 h (Fig. 2A). Notably, immunoblot analysis of TAp63α using BN-PAGE revealed that CDDP treatment did not induce TAp63α tetramerization, unlike 0.45 Gy irradiation for 2 h (Fig. 2B). Consistent with these observations, cells transfected with TAp63α and treated with 20 μM CDDP showed expression of Cleaved CASP3, an apoptotic marker, and exhibited a higher percentage of Cleaved CASP3-positive cells (Fig. 2C, D).

To further validate that tetramer formation is unnecessary for apoptotic induction, we examined three TAp63α mutant plasmid constructs-generously provided by Dr. Volker Dötsch-with mutations in the α-helix of the tetramerization domain (TD), previously analyzed by Dr. Dötsch’s group in the p21 promoter assay [13]. Due to these mutations, the plasmid constructs lacked binding activity on the p21 promoter (Fig. 2E). Cells transfected with these mutants and treated with 20 μM CDDP also displayed apoptosis levels comparable to those with wild-type TAp63α (Fig. 2F, red arrowheads). Importantly, these mutants did not form tetramers following 20 μM CDDP treatment, in contrast to the 0.45 Gy irradiation group (Fig. 2G), suggesting that CDDP induces apoptosis through TAp63α dimers.

### CDDP induces TAp63α accumulation in oocytes of primordial follicles as an early critical response prior to activation

**Fig. 3:**
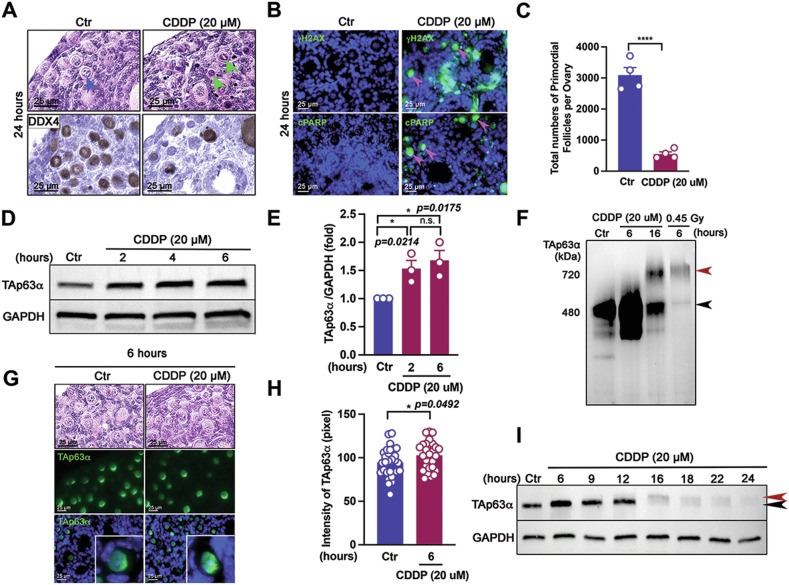
CDDP induces the accumulation of TAp63α in oocytes of primordial follicles ex vivo without tetramer formation. **A** Histology and DAB staining for Dead-box helicase 4 (DDX4), an oocyte marker [26], in ovaries treated with solvent (Control, Ctr) or 20 μM CDDP for 24 h. Intact primordial follicles and apoptotic ones are indicated by blue and green arrowheads, respectively. **B** Expression of γH2AX, a DNA damage marker, and cPARP, an apoptotic marker, in ovaries cultured with either solvent (Control, Ctr) or 20 μM CDDP for 24 h. Pink arrowheads indicate the signals. **C** Total numbers of primordial follicles per ovaries (*n* = 4). **D** Immunoblot analysis showing time-dependent expression of TAp63α and GAPDH. **E** Quantification of TAp63α intensity, expressed as fold changes. Each dot represents a biologically distinct female ovary (*n* = 3 in each group). **F** Immunoblot analysis for TAp63α on BN-PAGE using ovaries treated with solvent (Control, Ctr) and 20 μM CDDP, harvested at 6 and 16 h. An ovary exposed to 0.45 Gy irradiation for 4 h was used as a positive control. The molecular weights of the TAp63α dimer (480 kDa, a black arrowhead) and tetramer (720 kDa, a red arrowhead) are indicated on the BN-PAGE. **G** Histology and immunofluorescence assay of TAp63α in ovaries cultured with solvent (Control, Ctr) or 20 μM CDDP for 6 h. Insets show TAp63α expression in oocytes of primordial follicles. **H** Intensity analysis of TAp63α in primordial follicles (*n* = 30) at 6 h post-CDDP treatment. **I** Immunoblot analysis of TAp63α and GAPDH at each time point. Phosphorylated and unphosphorylated bands on SDS-PAGE are marked with red and black arrowheads, respectively. Statistical significance: n.s. not significant; *p < 0.05; ****p < 0.0001.

CDDP was selected to study the dynamics of TAp63α due to its effect on TAp63α accumulation, thus avoiding the complexities associated with CPA’s metabolic activation. To determine whether CDDP directly causes TAp63α accumulation in oocytes of primordial follicles, ovaries were cultured ex vivo and treated with two doses of CDDP (Fig S3A). Culturing PD5 mouse ovaries for 24 h with 20 µM of CDDP induced follicular damage (Fig. 3A), as evidenced by condensed nuclei in the oocytes of primordial follicles (green arrowheads). This treatment led to a significant loss of primordial and primary follicles, though secondary follicles remained unaffected, as indicated by a reduced number of DDX4-positive oocytes (Figs. 3A and S3A). Apoptosis was progressed in the primordial follicle oocytes, as shown by increased expression of γH2AX, a DNA damage marker (Fig. 3B top panel), and Cleaved PARP (cPARP), an apoptotic marker (Fig. 3B, bottom panel) [18]. Consequently, the total number of primordial follicles was significantly decreased compared to the control group at 24 h (Fig. 3C). Immunoblot analysis of ovary lysates confirmed a rapid accumulation of TAp63α protein at 2 h post-treatment in the 20 µM CDDP group (Fig. S3B), which persisted for up to 6 h (Fig. 3D, E). As demonstrated in the ex vivo model, TAp63α protein rapidly accumulated approximately 4 h after a 5 mg/kg CDDP injection (Fig. S1). CDDP at 20 µM significantly induced the accumulation of 480 kDa dimers without phosphorylation or tetramerization of TAp63α in the oocytes of primordial follicles at 6 h. However, the dimers weakened at 16 h, with some tetramers forming, while 0.45 Gy radiation exhibited strong tetramer bands (Fig. 3F). Immunofluorescence analysis further confirmed that TAp63α intensity increased at 6 h following treatment with 20 µM CDDP (Fig. 3G, H). Interestingly, TAp63α intensity began to decrease after 9 h and was nearly undetectable by 24 h, with faint phosphorylation bands observed at all time points on SDS-PAGE (Fig. 3I). These findings suggest that CDDP initially triggers TAp63α accumulation, followed by phosphorylation, without promoting tetramerization.

### CDDP-induced oocyte apoptosis is suppressed by inhibiting TAp63α protein synthesis

**Fig. 4:**
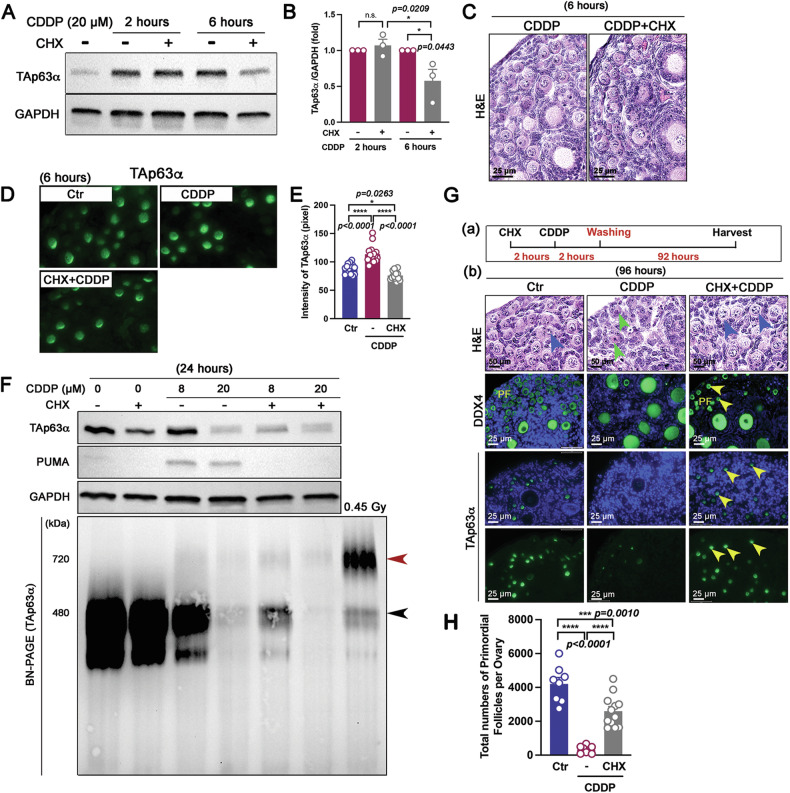
TAp63α is synthesized in response to CDDP leading to the loss of primordial follicles. **A** Immunoblot analysis of TAp63α and GAPDH at 2 and 6 h following treatment with CDDP, with or without pretreatment with cycloheximide (CHX). **B** Relative expression of TAp63α. Each dot represents a biologically distinct female ovary (*n* = 3 per group). **C** Histology of ovaries at 6 h post-exposure to CDDP or CDDP pretreated with CHX. **D** Immunofluorescence assay for TAp63α in ovaries. **E** Intensity analysis of TAp63α in oocytes of primordial follicles at 6 h (*n* = 3 mice per group). **F** Immunoblot analysis of TAp63α, PUMA, and GAPDH on SDS-PAGE, and TAp63α on BN-PAGE using ovarian extracts. An ovary from mice exposed to 0.45 Gy irradiation was used as a positive control for tetramer formation. The molecular weights of the TAp63α dimer (480 kDa, a black arrowhead) and tetramer (720 kDa, a red arrowhead) are indicated on the BN-PAGE. **G** (a) Schematic of ovarian culture ex vivo. (b) Histological analysis of ovaries and immunofluorescence assay showing DDX4 and TAp63α. In H&E staining, intact primordial follicles and apoptotic ones are indicated by blue and green arrowheads, respectively. The middle and bottom images highlight the expression of DDX4 and TAp63α, respectively. Primordial follicles (PF) in immunofluorescence assay were marked by yellow arrowheads. **H** Total number of primordial follicles per ovaries after 96 h of culture (*n* = 8 for Ctr and CDDP groups; *n* = 12 for CHX + CDDP group). Statistical significance: n.s., not significant; *p < 0.05; ***p < 0.001; ****p < 0.0001.

To investigate whether the CDDP-induced accumulation of TAp63α in oocytes of primordial follicles is regulated at the translational level, ovaries were co-treated with CDDP and cycloheximide (CHX), a translation inhibitor. Compared to ovaries treated with CDDP alone, those treated with both CDDP and CHX had significantly reduced expression levels of TAp63α protein at 6 h (Fig. 4A, B). Histological analysis during this period revealed intact and healthy follicles, suggesting that the reduction in TAp63α expression was primarily due to its suppressed de novo synthesis rather than the follicular damage (Fig. 4C). Immunofluorescence assays showed significantly lower TAp63α intensity in oocytes from the CDDP + CHX group compared to the CDDP alone group (Fig. 4D, E). PUMA, an apoptotic executive protein, has been established to contribute to primordial follicle oocyte apoptosis in response to DNA damage [22, 23]. Our results showed that PUMA was detected only in the ovaries trated with CDDP alone but not in ovaries treated with CDDP + CHX at 24 h (Fig. 4F), suggesting that the accumulation of TAp63α without hyper-phopsphorylation is sufficient to trigger apoptosis (Fig. 4F). The BN-PAGE data indicate that TAp63α synthesis was suppressed at 8 µM and completely inhibited at 20 µM by CHX treatment. Hyperphosphorylation was not induced under any condition, except for 0.45 Gy irradiation for 4 h, which did result in hyperphosphorylation (Fig. 4F). To minimize CHX toxicity on protein synthesis, ovaries were pre-treated with CHX for 2 h prior to CDDP exposure, which corresponds to the timing of TAp63α increase (Fig. S3B). After washing, the combined effects on primordial follicle survival were assessed (Fig. 4G-a). This finding was corroborated by histological analysis, immunofluorescence assays, and follicle quantification, all of which demonstrated that co-treatment with CHX and CDDP effectively prevented the loss of primordial follicles without affecting primary and secondary follicles, as confirmed by DDX4 and TAp63α immunofluorescence (Figs. 4G-b, H and S4A). These findings suggest a causal relationship between CDDP-induced TAp63α accumulation and apoptosis in oocytes of primordial follicles.

### Ubiquitin-mediated degradation of TAp63α occurs in oocytes within primordial follicles

**Fig. 5:**
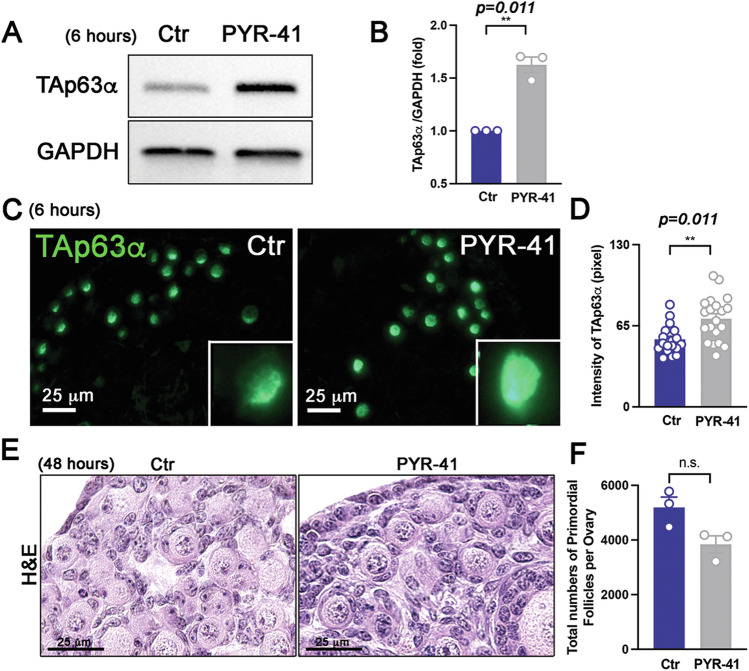
Ubiquitin-mediated degradation of TAp63α occurs in oocytes within primordial follicles. **A** Immunoblot analysis of TAp63α and GAPDH in ovaries cultured with solvent (Control, Ctr) or PYR-41 for 6 h. **B** Relative intensity of TAp63α. Each dot represents a biologically distinct female ovary (*n* = 3 per group). **C** Immunofluorescence assay of TAp63α in ovaries. **D** Intensity analysis of TAp63α in oocytes (*n* = 20) of primordial follicles from ovaries post-culture for 6 h (*n* = 3). **E** Histology of ovaries 48 h after exposure to PYR-41. **F** Total number of primordial follicles per ovaries following a 48-h culture period. Statistical significance: n.s. not significant; **p < *0.01*.

Previous research indicates that TA isoforms, particularly TAp63α, are rapidly degraded with a half-life of about one hour in vitro, making it one of the most swiftly degraded variants [19]. The E3 ubiquitin ligase ITCH has been shown to regulate and promote the degradation of TAp63α in the epidermis and primary keratinocytes in vitro [24]. It is also established that the active form of TAp63α is more susceptible to degradation than the inactive form in vitro [25]. However, the half-life of TAp63α in oocytes has not yet been reported. To explore the role of ubiquitination in regulating the dynamics of TAp63α in oocytes within primordial follicles, ovaries were treated with PYR-41, a ubiquitin-activating enzyme E1 inhibitor, for 6 h. Immunoblotting analysis of ovary lysates showed that PYR-41 significantly increased TAp63α accumulation (Figs. 5A, B, and S5A). This suggests that TAp63α undergoes rapid turnover through ubiquitin-dependent proteasomal degradation. Immunofluorescence assays further demonstrated significantly higher TAp63α expression levels in oocytes of primordial follicles in the PYR-41-treated groups compared to the control (Ctr) group (Fig. 5C, D). Ovaries treated with PYR-41 retained surviving primordial follicles after early increases in TAp63α accumulation (Fig. 5E, F). These findings suggest that TAp63α in oocytes of primordial follicles undergoes dynamic synthesis and degradation.

### Oocyte death in primordial follicles is induced by phosphorylation-related activation rather than stabilization of TAp63α by CDDP

**Fig. 6:**
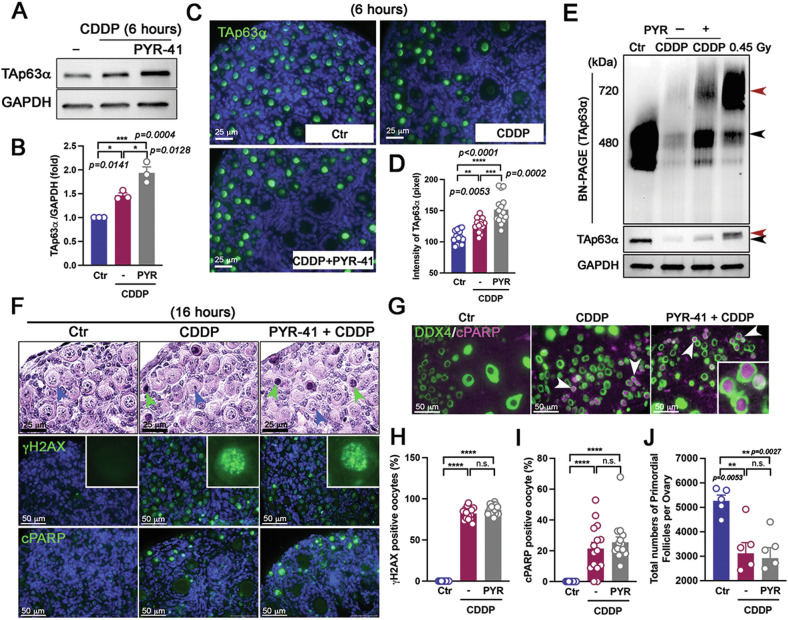
Oocyte death in primordial follicles requires phosphorylation-induced activation by CDDP treatment. **A** Immunoblot analysis of TAp63α and GAPDH. **B** Relative intensity of TAp63α. Each dot represents a biologically distinct female ovary (*n* = 3 per group). **C** Immunofluorescence assay of TAp63α in ovaries. **D** TAp63α intensity in oocytes of primordial follicles measured 6 h post-culture with solvent, CDDP, and CDDP + PYR-41. **E** Immunoblot analysis of TAp63α on BN-PAGE and of TAp63α and GAPDH on SDS-PAGE using ovarian extracts. An ovary from mice exposed to 0.45 Gy irradiation was used as a positive control for tetramer formation. The molecular weights of the TAp63α dimer (480 kDa, a black arrowhead) and tetramer (720 kDa, a red arrowhead) are indicated on the BN-PAGE. Phosphorylated and unphosphorylated bands on SDS-PAGE are marked with red and black arrowheads, respectively. **F** Histology of ovaries and expression of γH2AX and cPARP. In H&E staining, intact primordial follicles and apoptotic ones are indicated by blue and green arrowheads, respectively. **G** Immunofluorescence assay of cPARP expression and DDX4, an oocyte marker, in primordial follicles. Primordial follicles co-expressing cPARP and DDX4 are marked with white arrowheads. **H** Percentage of γH2AX-positive oocytes. **I** Percentage of cPARP-positive oocytes (*n* = 15 images from *n* = 3 ovaries in each group). **J** Quantification of primordial follicles in ovaries (*n* = 5 per group). Statistical significance: n.s. not significant; *p < 0.05; **p < 0.01; ***p < 0.001; ****p < 0.0001.

To determine whether co-treatment of ovaries with CDDP and PYR-41 leads to a greater increase in TAp63α accumulation, ovaries were cultured with both agents for 6 h. Western blot results and immunofluorescence analyses showed much higher TAp63α accumulation in oocytes of primordial follicles from the co-treatment group compared to the CDDP alone group (Fig. 6A–D), suggesting an increase in TAp63α due to translational upregulation and decreased degradation at this time point. However, the BN-PAGE immunoblot assay revealed that co-treatment with PYR-41 and CDDP preserved TAp63α at 16 h compared to the CDDP-only group, predominantly in its dimer form (indicated by the black arrowhead), rather than promoting tetramerization, as observed with hyperphosphorylation induced by 0.45 Gy irradiation for 4 h (indicated by the red arrowhead) (Fig. 6E). To explore whether the increased TAp63α expression, enhanced by PYR-41, accelerates oocyte death induced by CDDP, ovaries were cultured with either CDDP alone or CDDP combined with PYR-41. Histological analysis and immunostaining for DNA damage marker γH2AX and apoptotic markers cPARP revealed comparable levels of apoptosis in oocytes of primordial follicles at 16 h between the CDDP-alone group and the PYR-41 plus CDDP co-treatment group (Fig. 6F). Both groups exhibited similar levels of γH2AX foci in the oocytes of primordial follilces (insets in Fig. 6F). Double staining for DEAD-box helicase 4 (DDX4, an oocyte marker) [26–28] and cPARP showed that apoptosis primarily occurred in the oocytes of primordial follicles (white arrowheads; inset in Fig. 6G). Most oocytes in primordial follicles exhibited γH2AX signals at 16 h (82.11% in the CDDP-only group and 86.52% in the CDDP + PYR-41 group). A subset of these oocytes were cPARP-positive at this time point (21.43% in the CDDP-only group and 25.50% in the CDDP + PYR-41 group), suggesting that some primordial follicles had already undergone apoptosis. The percentages of γH2AX and cPARP were elevated in both the CDDP-alone and combination treatment groups compared to the control group (Fig. 6H, I), whereas BAX expression was notably higher in the CDDP + PYR41 group than in the CDDP-only group (Fig. S5B). Follicle counts showed similar numbers of surviving primordial follicles across treatment groups at this time point (Fig. 6J). These findings indicate that oocyte death is driven more by CDDP-induced phosphorylation than by the increased translational stabilization of TAp63α.

### TAp63α accumulation is controlled at the initiating step before activation through CHK2

**Fig. 7:**
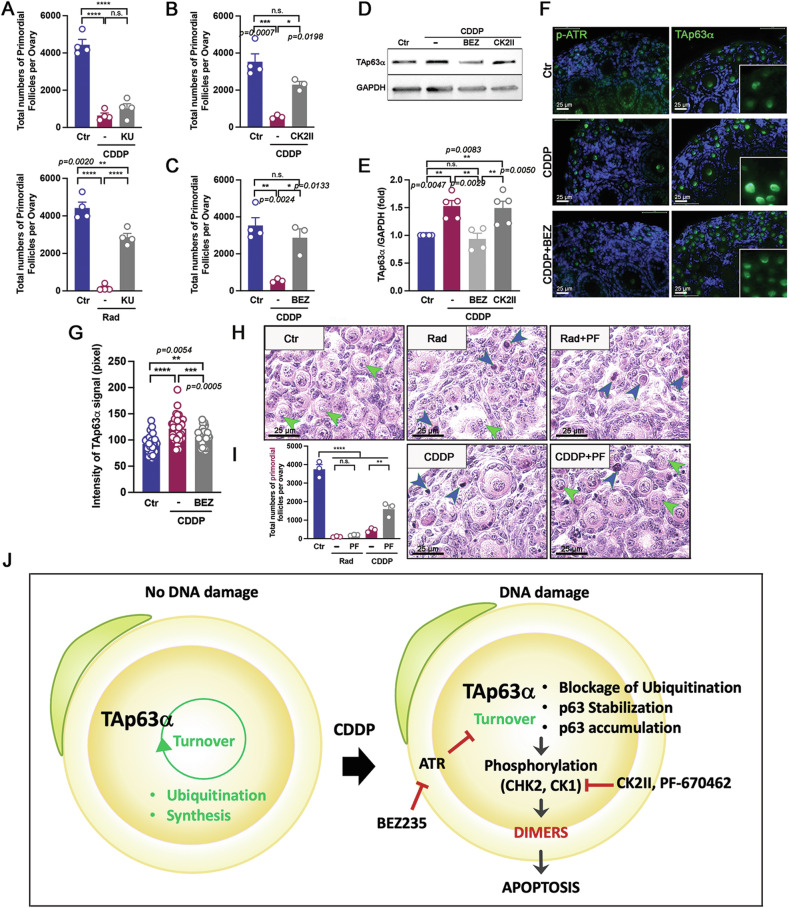
TAp63α accumulation is regulated at the initiating step before activation through CHK2 and CK1. **A–C** Quantification of primordial follicles in ovaries treated with 20 µM ATM inhibitor (KU-55933), 20 µM CHK2 inhibitor (CK2II), and 20 µM ATR inhibitor (BEZ235) in response to 20 µM CDDP or 0.45 Gy irradiation. **D** Immunoblot analysis of TAp63α and GAPDH in ovaries cultured with 20 µM BEZ235 or 20 µM CK2II for 6 h. **E** Quantification of TAp63α and GAPDH in immunoblots. **F**, **G** Immunofluorescence assay of p-ATR and TAp63α, along with TAp63α intensity at 6 h post-treatment. **H**, **I** Histological analysis and quantification of primordial follicles in the ovaries 24 h post-exposure to 0.45 Gy irradiation, 0.45 Gy irradiation+25 µM PF-670462, 20 µM CDDP, or 20 µM CDDP + 25 µM PF-670462. Intact primordial follicles and apoptotic ones are indicated by green and blue arrowheads, respectively. **J** Graphic summary of TAp63α turnover in intact oocytes and the stabilization and activation cascade of TAp63α following DNA damage detection. Statistical significance: n.s. not significant; *p < 0.05; **p < 0.01; ***p < 0.001; ****p < 0.0001.

Previous studies have shown that CDDP treatment preferentially activates Ataxia Telangiectasia and Rad3-related (ATR) kinase over ataxia telangiectasia mutated (ATM) kinase [3]. This activation cascade involves CHK2 and CK1, which phosphorylate TAp63α [3, 12, 17, 29, 30]. Our results indicate that the ATR inhibitor (BEZ235) and the CHK2 inhibitor (CK2II) [12] suppressed CDDP-induced oocytes apoptosis and primordial follicles loss, while the ATM inhibitor (KU-55933) did only for 0.45 Gy irradiation-induced apoptosis (Fig. 7A–C). Additionally, our results demonstrated that while the ATR inhibitor (BEZ235) suppressed the synthesis of TAp63α in primordial follicles, the CHK2 inhibitor (CK2II) did not affect TAp63α accumulation (Fig. 7D, E). This was further corroborated by the observed decrease in TAp63α intensity in the CDDP + BEZ235 group compared to CDDP-only group (Fig. 7F, G). To determine whether TAp63α undergoes further phosphorylated by Casein kinase 1 (CK1) despite not forming tetramers, we evaluated the effect of the CK1 inhibitor PF-670462 on primordial follicles. Interestingly, PD-670462 effectively blocked cisplatin-induced oocyte death but did not protect oocytes from radiation-induced damage. This suggests that CK1-mediated phosphorylation plays a role in TAp63α activation upon exposure to CDDP, even in the absence of tetramer formation (Fig. 7H–I). In the absence of DNA damage, TAp63α undergoes continuous turnover through ubiquitination and synthesis. However, upon exposure to CDDP-induced DNA damage, ATR is activated, likely altering TAp63α turnover, which leads to its stabilization and accumulation. Following stabilization, TAp63α is phosphorylated by CHK2 and CK1, leading to its activation (Fig. 7J). Once activated, TAp63α primarily exists as a dimer, which subsequently induces apoptosis (Fig. 7H). Furthermore, we observed that carboplatin, another alkylating agent, also promotes TAp63α accumulation (Fig. S5C), suggesting a similar mechanism in the oocytes of primordial follicles as that induced by CDDP.

## Discussion

Our previous study identified a distinct apoptotic pathway induced by CDDP in oocytes of primordial follicles compared to irradiation, highlighting differences in the phosphorylation of TAp63α [3, 17, 31, 32]. Here, we demonstrated for the first time that CDDP causes an accumulation of TAp63α protein within primordial follicle oocytes. This increase is largely due to a shift in the balance between degradation and synthesis, favoring new synthesis and stabilization through the blockage of degradation. The accumulated TAp63α is then phosphorylated by CHK2 and CK1, playing a key role in apoptotic pathway. However, the activated TAp63α does not form tetramers, as evidenced by strong dimer bands in BN-PAGE.

TAp63α is highly expressed in primary oocytes arrested in the prophase of meiosis I [33]. It is maintained in an inactive, dimeric status but is ready for rapid activation upon DNA damage through unfolding its inhibitory structure. According to a previous study, the activation of TAp63α-mediated apoptotic pathway does not require additional translation in oocytes of primordial follicles [13]. However, our results show that TAp63α levels increased upon CDDP treatment. Additionally, suppressing TAp63α accumulation induced by CDDP had a protective effect on primordial follicle oocytes, underscoring the a crucial role of TAp63α accumulation in initiating the apoptotic pathway. While the active form of TAp63α is more prone to degradation, as evidenced by a significant decrease in intracellular TAp63α at 17 h post-CDDP treatment, the inactive forms can accumulate to much higher concentrations [13, 25].

The mechanism of TAp63α accumulation involves the stabilization of intracellular TAp63α, which is dependent on its structure and regulated by ubiquitination-dependent mechanisms [34, 35]. As shown in the literature, substantial accumulation of TAp63α was observed in oocytes of primordial follicles when an ubiquitination inhibitor was used. The Hect (homologous to the E6-associated protein C terminus)-containing Nedd4-like ubiquitin protein ligase (ITCH) has been shown to influence p63α protein levels [24]. ITCH binds, ubiquitylates, and promotes the degradation of p63 through the physical interaction between the PY (Y504F for TAp63) and the SAM (sterile a motif) domains of p63 in primary mouse keratinocytes. Another study demonstrated that TAp63α is regulated by proteasome degradation, showing that Cyclin-dependent kinase 5 and Abl enzyme substrate 1 (Cables1) binds to the TAp63α isoform and stabilizes it from proteasomal degradation [36]. Moreover, TAp63α concentration has been shown to decrease through sumoylation-mediated degradation of the dimeric form of TAp63α [36, 37]. These findings indicate that multiple regulatory pathways modulate the stability of TAp63α in the oocytes of primordial follicles.

Another mechanism of TAp63α accumulation is its de novo synthesis in response to DNA damage. Inhibiting the synthesis of TAp63α resulted in a significant reduction in its levels 6 h following treatment with CDDP. A previous study also reported that treatment with CHX reduced the expression of TAp63α in oocytes of mouse primordial follicles [13]. These results suggest that de novo synthesis of TAp63α occurs in oocytes within primordial follicles and is crucial for replenishing its levels after endogenous degradation. The ability of oocytes to synthesize TAp63α post-degradation indicates a dynamic regulatory mechanism, underscoring the importance of TAp63α synthesis in maintaining the cellular response to gonadotoxic agents. This insight highlights the intricate balance between the degradation and synthesis of TAp63α, depending on the stress conditions in oocytes within primordial follicles.

It has been accepted that all three p53 family members-p53, p63, and p73-form tetramers in their active forms [38, 39]. Previous studies suggested the necessity of hyperphosphorylation and tetramerization of TAp63α in triggering primordial follicle oocyte apoptosis following chemotherapy. However, our results demonstrate that these processes are not always essential for inducing oocyte death. When oocytes are exposed to CDDP and CPA, the stabilization and accumulation of TAp63α are necessary. Although TAp63α was phosphorylated in response to CDDP and CPA ex vivo, hyperphosphorylation was less prominent than that seen in the irradiation-treated group. This may be because TAp63α activation involves phosphorylation of only a limited number of residues. We also found that the hyperphosphorylation of TAp63α does not occur even in the presence of a ubiquitination inhibitor. Examining the increase in TAp63α levels after treatment with CDDP and a ubiquitination inhibitor revealed that this elevation is crucial, implying that reaching and surpassing this level of increase is necessary. However, it is assumed that TAp63α levels are not the sole trigger of apoptosis. We previously discovered that apoptosis of primordial follicle oocytes can be induced by the formation of heterotetramers between TAp63α and TAp73α proteins, particularly since TAp73α is also upregulated by CDDP treatment [3, 13, 40]. However, the current study proved that CDDP and CPA induce apoptosis without the tetramer formation of TAp63α.

The DNA damage response by CDDP treatment initiates the activation of ATR before the activation of TAp63α [3]. Our results showed a decrease in TAp63α accumulation with the pharmacological inhibition of ATR but not CHK2, suggesting that the stabilization of TAp63α occurs before phosphorylation, indicating a potentially novel mechanism by which ATR regulates TAp63α accumulation.

We acknowledge the limitations of this study. First, we cannot rule out the possibility that tetramerization of even a small portion of TAp63α may be sufficient to propagate apoptotic signals and determine oocyte fate when primordial follicles are exposed to CDDP, which may differ from the mechanism of radiation-induced oocyte death in primordial follicles. While we are currently unable to explain this phenomenon where TAp63α dimers appear to induce oocyte apoptosis, this is beyond the scope of our current study. Nonetheless, we emphasize that TAp63α responds differently to CDDP-induced DNA damage compared to radiation-induced DNA damage. Second, we aimed to demonstrate that TAp63α expression is reduced in the CHX + CDDP group compared to the CDDP group, leading to decreased expression of PUMA, as shown in Fig. 4. Since PUMA is a downstream target of TAp63α, its expression may be more influenced by the absence of TAp63α than by the direct effects of CHX. This suggests that TAp63α plays a central role in driving oocyte death. However, as cycloheximide (CHX), a translation inhibitor, can also block the synthesis of other key proteins involved in oocyte apoptosis. Thus, it is challenging to distinguish the specific effects of CHX on the synthesis of TAp63α versus PUMA in this experiment. Third, TAp63α expression is increased in the PYR-41 group compared to the CDDP group, as shown in Fig. 6A. This indicates that TAp63α is also regulated by PYR-41. However, PYR-41, an E1 enzyme inhibitor, affects the degradation of various proteins, including TAp63α. Despite these limitations, we propose that CDDP induces oocyte apoptosis via a TAp63α-dependent pathway that differs from the response to radiation-induced DNA damage.

In conclusion, our study demonstrates that upon CDDP-induced DNA damage in oocytes of primordial follicles, TAp63α is initially stabilized before undergoing phosphorylation-induced activation. This process ultimately triggers the apoptotic pathway, leading to oocyte death. Further investigations are needed to identify the upstream regulators of TAp63α stabilization as well as determine how CDDP induces oocyte apoptosis via the dimeric but not the tetrameric form of TAp63α.

## Materials and methods

### Animal

CD-1® IGS mice were purchased from Charles River Laboratories. All mouse-related procedures were approved by the Institutional Animal Care and Use Committee (IACUC) at the University of Nebraska Medical Center (UNMC). The mice were housed in the Comparative Medicine facilities at UNMC, with access to food and water *ad libitum*. Environmental conditions such as temperature, humidity, and a photoperiod of 10 h of light and 14 h of darkness were constantly maintained. The birth date was designated as postnatal day 0 (PD0), and male pups were euthanized on PD0 to maintain consistent body weight among female pups. Female CD-1 pups at PD5 were intraperitoreally administrated injections of either solvent (100 μL), 5 mg/kg Cis-Platinum (II) Diammine Dichloride (cisplatin, CDDP, P4394, Sigma Aldrich, MO, USA), or 150 mg/kg cyclophosphamide (CPA, 111017846, Fisher Scientific, MA, USA), and subsequently placed with foster moms until their ovaries were harvested at specific time intervals. Animals were randomly assigned to treatment groups; however, other procedures, such as immunofluorescence assays and BN-PAGE gel analysis, were conducted without randomization due to specific treatment performed. The sample size for the animal study was determined based on prior research, practical considerations, and ethical guidelines to minimize animal use while ensuring adequate data collection. We were not blinded to group allocation during the experiment or outcome assessment. Harvested ovaries were allocated to distinct experimental groups, and uniform-sized pups were injected with different treatments (CPA or CDDP). The ovaries were subsequently used for further evaluation.

### Whole organ ovary culture ex vivo

The whole ovarian organ culture procedure was performed according to previously established methods [3, 17]. Ovaries collected from PD5 CD-1 female mice were carefully dissected from the ovarian bursa in a dissection medium. Ovaries were then placed on 0.4 μm Millicell cell culture inserts (PICM01250, MilliporeSigma, MA, USA) in a medium containing either an inhibitor or vehicle for a pre-treatment duration of 2 h, followed by exposure to 20 µM CDDP. We also used a dose of 0.45 Gy irradiation using the RS 2000 Small Animal Irradiator (Rad Source Technologies, GA, USA) and cultured the ovaries for 2, 4, and 6 h, as described in previous studies and applied for hyperphorylation of TAp63α [3]. Cultures were maintained at 37 °C in a 5% CO_2_ incubator until collection. The concentration of 20 μM CDDP was selected based on the peak blood concentration observed in humans undergoing treatment with CDDP [41, 42]. The culture medium comprised minimum essential medium α (MEM-α, 32571036, Gibco, MA, USA) supplemented with 3 mg/ml bovine serum albumin (BSA, BP9703100, ThermoFisher Scientific, MA, USA) and 5 μg/ml insulin-transferrin-selenium (ITS, 41400045, Gibco). The CDDP solution was freshly prepared each time using Dulbecco’s phosphate-buffered saline without calcium and magnasium (DPBS, no calcium, no magnesium, 14190144, ThermoFisher scientific). As CPA requires metabolic activation in the liver to exert its effects, in vitro studies necessitate the use of its metabolite, with 4-Hydroperoxy Cyclophosphamide (4-HC, one of CPA metabolites, 19527-1, Cayman Chemical, MI, USA) prepared in dimethyl sulfoxide (DMSO). Carboplatin (TCC2043-100MG) was purchased from VWR (Radnor, PA, USA). Inhibitors were obtained from various suppliers: 20 μM Chk2 inhibitor II hydrate (CK2II, C3742) from MilliporeSigma; 20 μM BEZ235 (S1009) and 20 μM KU-55933 (3544) from Selleck Chemicals (TX, USA), and 500 ng/ml Cycloheximide (CHX, C1988), 50 μM PYR-41 (662105), and 25 µM PF-670462 (SML0795) from Sigma Aldrich.

### Follicle counting

The ovaries were fixed in Modified Davidson’s Fixative (3600, EKI, IL, USA) for 24 h at 4 °C, then processed into paraffin blocks and serially sectioned at 5 μm thickness. For histological analysis, the sections were stained with hematoxylin and eosin (H&E). Following established protocols [3, 17, 43, 44], ovarian follicles were counted, and the quantity of follicles belonging to each class was determined through classification.

### Cell culture and transfection

The H1299 non-small cell lung carcinoma cell line (CRL-5803, ATCC, Manassas, VA) was cultured in RPMI-1640 medium (11879-020, Gibco) supplemented with 10% heat-inactivated FBS (NC1505355, ThermoFisher) and 1X penicillin–streptomycin (15140122, Gibco) at 37 °C in 5% CO_2_. At 70–80% confluence, cells were passaged using TrypLE™ Express (12605-010, Gibco) for 3 min at 37 °C. Cells in the logarithmic growth phase were selected for subsequent experiments and transfected with plasmids using TransFectin^TM^ Lipid Reagent (1703350, BIO-RAD, Hercules, CA), following the manufacturer’s protocol. After 24 h, cells were treated with 20 µM CDDP and exposed to 0.45 Gy irradiation, then harvested in 100 µl of cell lysis buffer for further analysis. Additionally, cells were irradiated at 0.45 Gy using the RS 2000 Irradiator (Rad Source Technologies, GA, USA) for 2 and 4 h.

### Immunoblotting

Immunoblotting was performed following established procedures. Protein lysates (8 μg/well) were loaded onto the 4–15% Mini-PROTEAN^®^ TGX^TM^ Precast Protein Gels (#4561084, Bio-Rad Laboratories, IL, USA) and transferred to a PVDF membrane using the Trans-Blot^®^ Turbo^TM^ Transfer System (#1704150, Bio-Rad Laboratories). Antibodies used for detection were as follows: p63-α (D2K8X) XP^®^ (13109 s) and glyceraldehyde 3-phosphate dehydrogenase (GAPDH, 5174) from Cell Signaling Technology (MA, USA), anti-p53 upregulated modulator of apoptosis (PUMA, ABC158) from MilliporeSigma, DEAD-box helicase 4 (DDX4, ab270534) from Abcam (Cambridge, United Kingdom), and MSY2, a member of the Y-box family of proteins solely expressed in male and female germ cells [45–47], a gift from Dr. Paula Stein at the National Institute of Environmental Health Sciences, NIH.

### Blue native PAGE

BN-PAGE analysis was performed following established protocols [3, 12]. Briefly, four ovaries per indicated condition were harvested in prepared lysis buffer, supplemented with 1x protease and phosphatase inhibitor cocktail (PPC1010, MilliporeSigma). The lysis was performed and the lysate was supplemented with 40 mM CHAPS (19899, MilliporeSigma) and 1 µl Benzonase^®^Nuclease (E1014, MilliporeSigma), followed by incubation. The resulting supernatant was loaded into NativePAGE^TM^ 3–12%, Bio-Tris, 1.0 mm, Mini Protein Gels (BN1001BOX, Invitrogen, MA, USA) according to the manufacturer’s instructions. The cathode buffer was supplemented with 0.002% Coomassie G250, and the separation was performed at 4 °C for 60 min at 150 V, followed by 60 min at 250 V. Proteins were then transferred to Invitrolon^TM^ PVDF/Filter Paper Sandwiches membrane (LC2005, Invitrogen). The Clarity Western ECL Substrate (#1705061, Bio-Rad Laboratories) was used for protein detection, and the iBright^TM^ CL1500 Imaging System (A44114, Invitrogen) was used for protein exposure. For native PAGE analysis of TAp63α expressed in H1299 cells, cells were treated and subsequently harvested after 24 h in 100 µl of cell lysis buffer containing 1× Native PAGE sample buffer, 1% DDM, 1× protease and phosphatase inhibitors cocktail (PPC1010, MilliporeSigma), and 0.5 µl Benzonase (E-1014, MilliporeSigma) per sample. Cells were lysed by pipetting the solution up and down several times. The remaining steps followed the protocol described above.

### Immunofluorescence assays and DAB staining

Immunofluorescence assay and DAB staining were performed following established procedures [3, 17, 31, 43]. The Primary antibodies, along with their catalog numbers and dilutions, were as follows: p63-α (D2K8X) (13109S), γH2AX (9718S), cleaved poly(ADP-ribose) polymerase (cPARP, Cleaved PARP, 9548S), and Cleaved CASP3 (9661S) from Cell Signaling Technology, DDX4 (ab270534) from Abcam, and BAX (p-19) (sc-526) from Santa Cruz Biotechnology Inc. DAPI (4′,6-diamidino-2-phenylindole) was used as a nuclear counterstain in fluorescence microscopy. The antibody reaction for p-ATR (2853, Cell Signaling) was enhanced using the Alexa Fluor 488 Tyramide SuperBoost Kit (B40932, Invitrogen). The Metal Enhanced DAB Substrate Kit (34065, ThermoFisher Scientific) was used for DAB (3,3′-diaminobenzidine) staining. The intensity of TAp63α signals was assessed using ImageJ, analyzing at least five random non-overlapping images captured under identical exposure conditions across multiple experiments (*n* ≥ 3). The specificity of secondary antibody binding was confirmed by the absence of signal in negative controls incubated without primary antibodies. All images were captured using the EVOS M7000 Imaging System (AMF700, Invitrogen).

### TUNEL assay

TUNEL staining was performed using the DeadEnd™ Fluorometric TUNEL System (G3250, Promega, Madison, WI), following the manufacturer’s instructions.

### Statistical analysis

Graphs were generated using Prism 10.2.3 software (GraphPad Software Version 9, Inc., CA, USA) and are presented as mean with ±SEM. Normality test and homogeneity of variances were assessed all samples. Multiple variable analyses were conducted using appropriate statistical tests. For direct comparisons between two groups, unpaired t-tests with Welch’s correction or Mann-Whitney test were utilized to evaluate mean differences between datasets. For comparisons involving more than two groups, ordinary one-way ANOVA with Tukey’s multiple comparisons test was used. *P* values less than 0.05 were considered statistically significant. In the notation used, n.s. represents not significant, and *, **, ***, and **** represent *p* < 0.05, *p* < 0.01, *p* < 0.001, and *p* < 0.0001, respectively.

## Supplementary information

SUPPLEMENTAL MATERIAL

## Data Availability

The data supporting the findings of this study are available from the corresponding author upon reasonable request.
