# Supplementary material for: Oocyte death is triggered by the stabilization of TAp63α dimers in response to cisplatin
Source: Cell Death Dis. 2024 Nov 7;15(11):799. doi: 10.1038/s41419-024-07202-7 (PMC11544165; doi:10.1038/s41419-024-07202-7)
Supplement: Supplementary file 1 — SUPPLEMENTAL MATERIAL [file 41419_2024_7202_MOESM1_ESM.docx]

**Supplementary Figures**

**
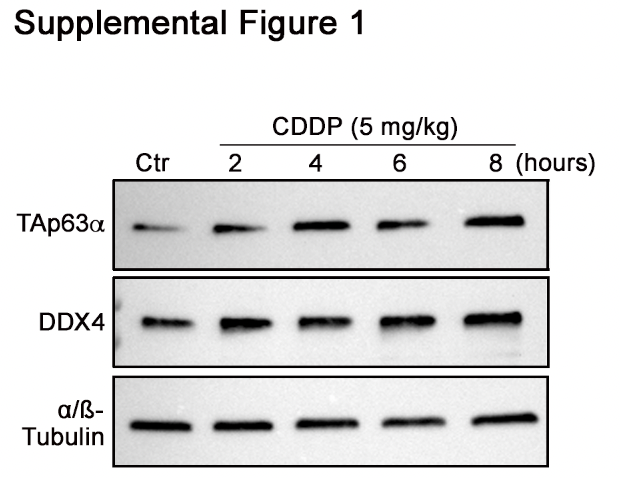
**

**Figure S1. Accumulation of TAp63α following exposure to CDDP.** Immunoblot analysis of the time-dependent expression of TAp63α, DDX4, and α/β-tubulin in ovaries from mice treated with 5 mg/kg CDDP.

**
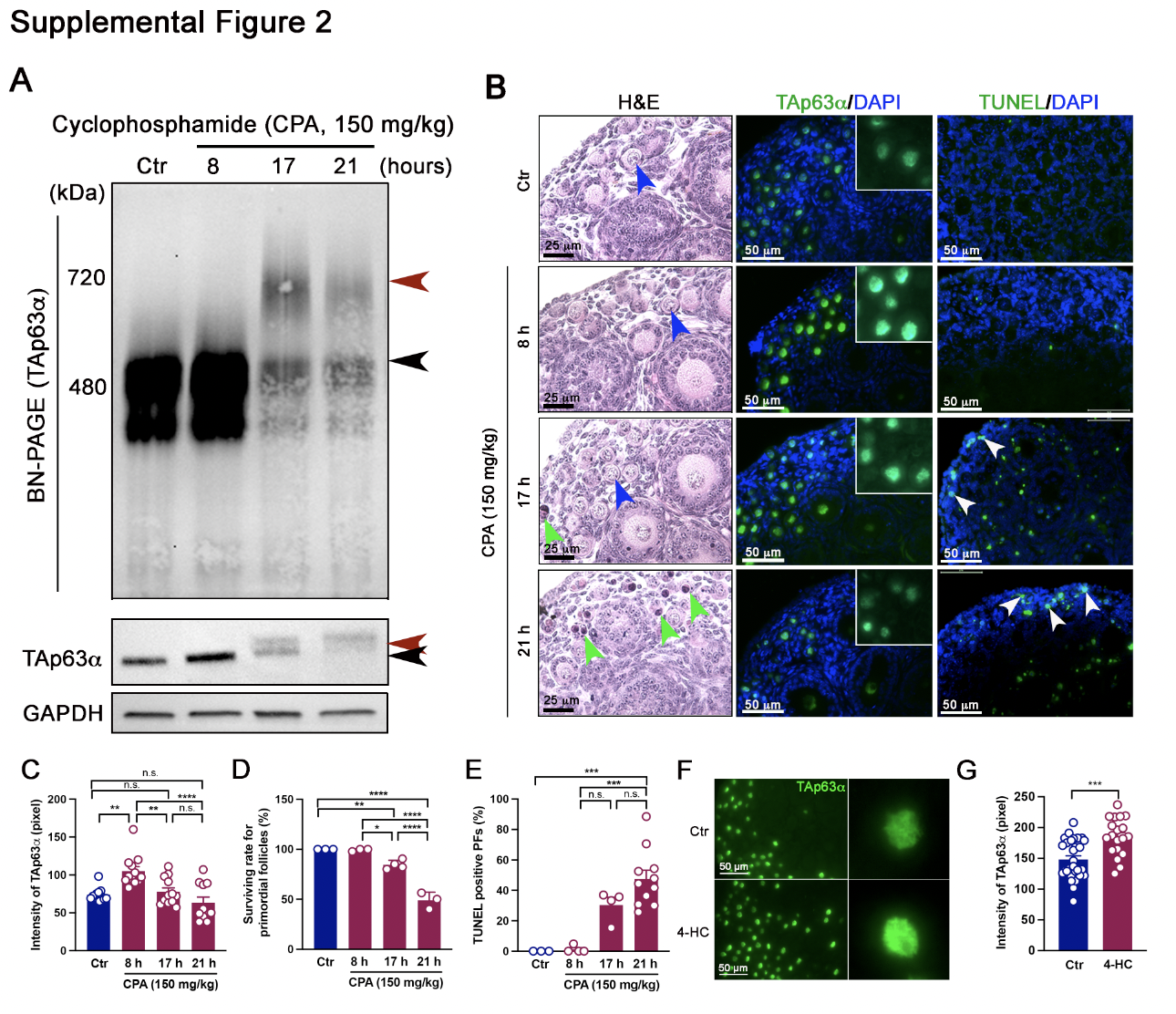
**

**Figure S2. TAp63α is accumulated in dimeric form following CPA injection.** **A**. Immunoblot analysis of TAp63α on BN-PAGE and TAp63α and GAPDH on SDS-PAGE using ovarian extracts from mice exposed to 150 mg/kg CPA. Ovaries were harvested at 0, 8, 17, and 21 hours post-exposure. The molecular weights of the TAp63α dimer (480 kDa, a black arrowhead) and tetramer (720 kDa, a red arrowhead) are indicated on the BN-PAGE. Phosphorylated and unphosphorylated bands on SDS-PAGE are marked with red and black arrowheads, respectively. **B.** Histological analysis with H&E staining, immunofluorescence assay for TAp63α, and TUNEL assay in ovarian samples from mice treated with either solvent (Control, Ctr) or 150 mg/kg CPA at 8, 17, and 21 hours post-injection. Intact primordial follicles are marked by blue arrowheads, and apoptotic ones by green arrowheads. Insets show TAp63α expression at each time point, with TUNEL-positive oocytes indicated by white arrowheads. **C.** Quantification of TAp63α Intensity. **D.** Survival rate of primordial follicles. **E.** Quantification of TUNEL-positive primordial follicles (PFs) in each ovarian samples. **F and G.** Expression and intensity of TAp63α signals in ovaries exposed to 4-HC *ex vivo* at 6 hours. Statistical significance: *n.s., not significant; *, p < 0.05; **, p < 0.01; ***, p < 0.001; ****, p < 0.0001.*

**
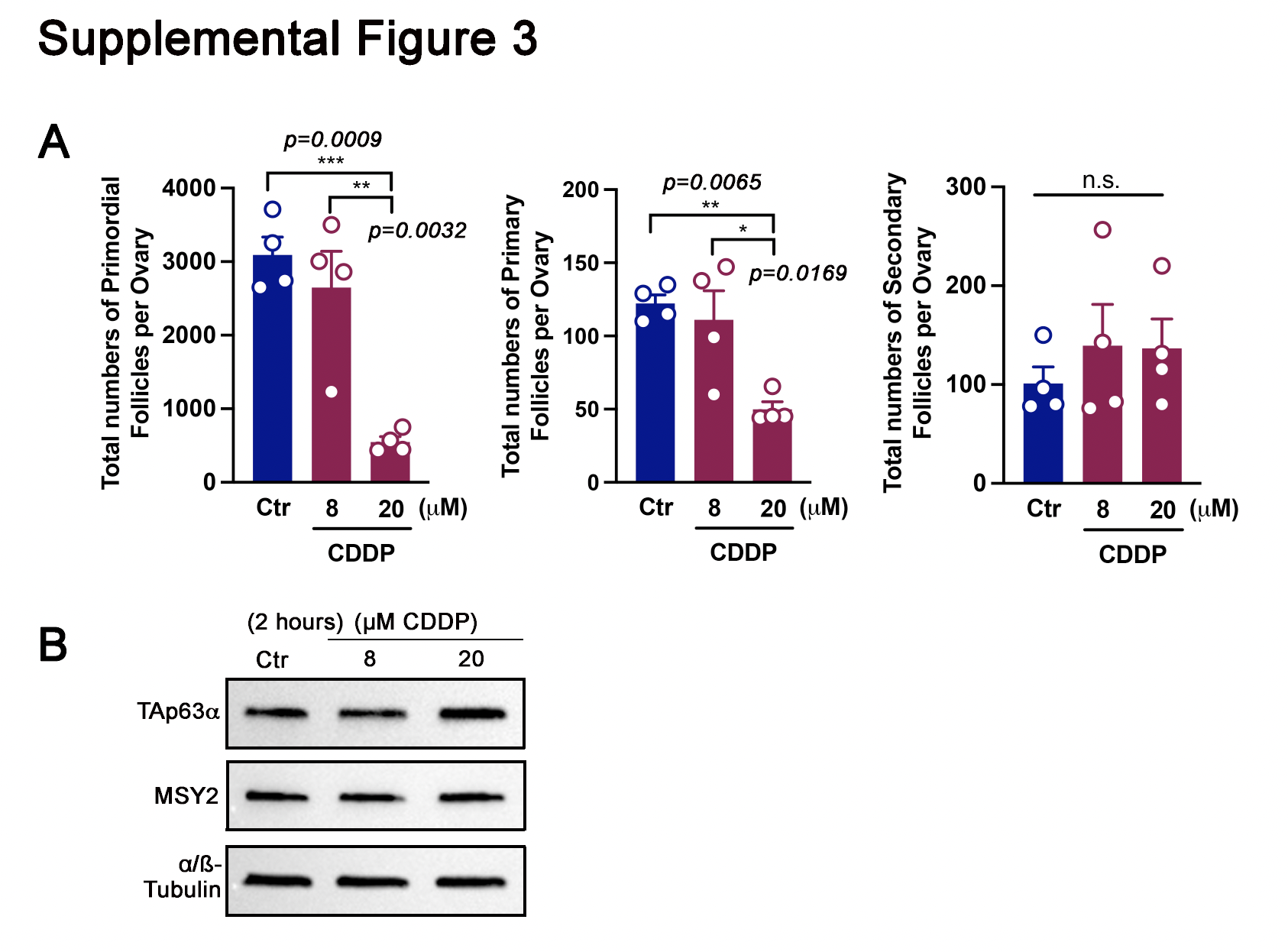
**

**Figure S3. A dose of CDDP sufficient to induce TAp63α expression results in the loss of primordial follicles. A.** Quantification of primordial, primary, and secondary follicles in ovaries exposed to 8 or 20 µM CDDP for 96 hours. **B.** Immunoblot assay for TAp63α, MSY2, and α/β-tubulin in ovaries treated with 8 or 20 µM CDDP for 2 hours. Statistical significance: *n.s., not significant; *, p < 0.05; **, p < 0.01; ***, p < 0.001.*

**
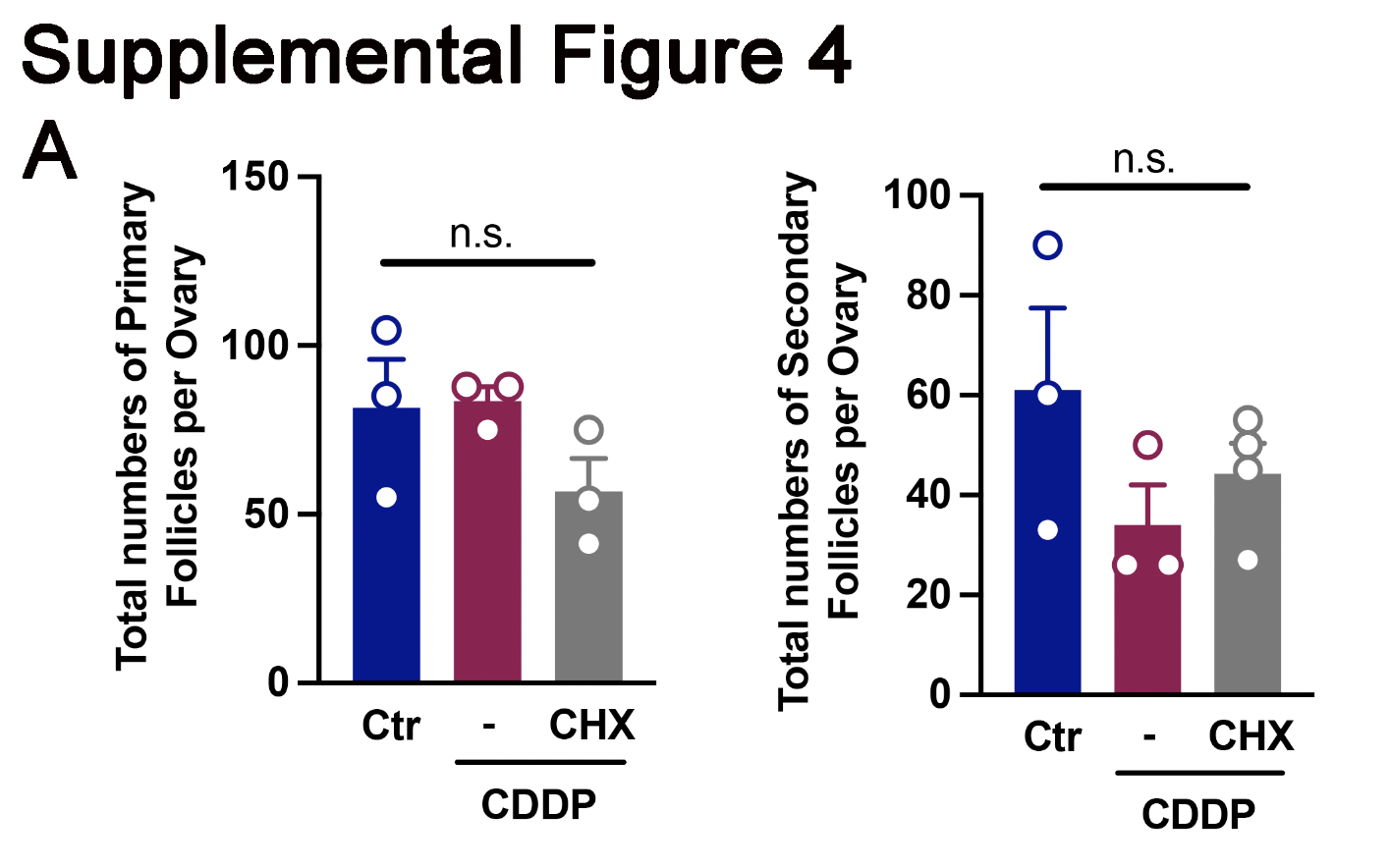
**

**Figure S4. Pretreatment with CHX does not alter the number of primary and secondary follicles.** **A.** Quantification of primary and secondary follicles in ovaries treated *ex vivo* with CDDP and CDDP+CHX for 96 hours. Statistical significance: *n.s., not significant.*

**
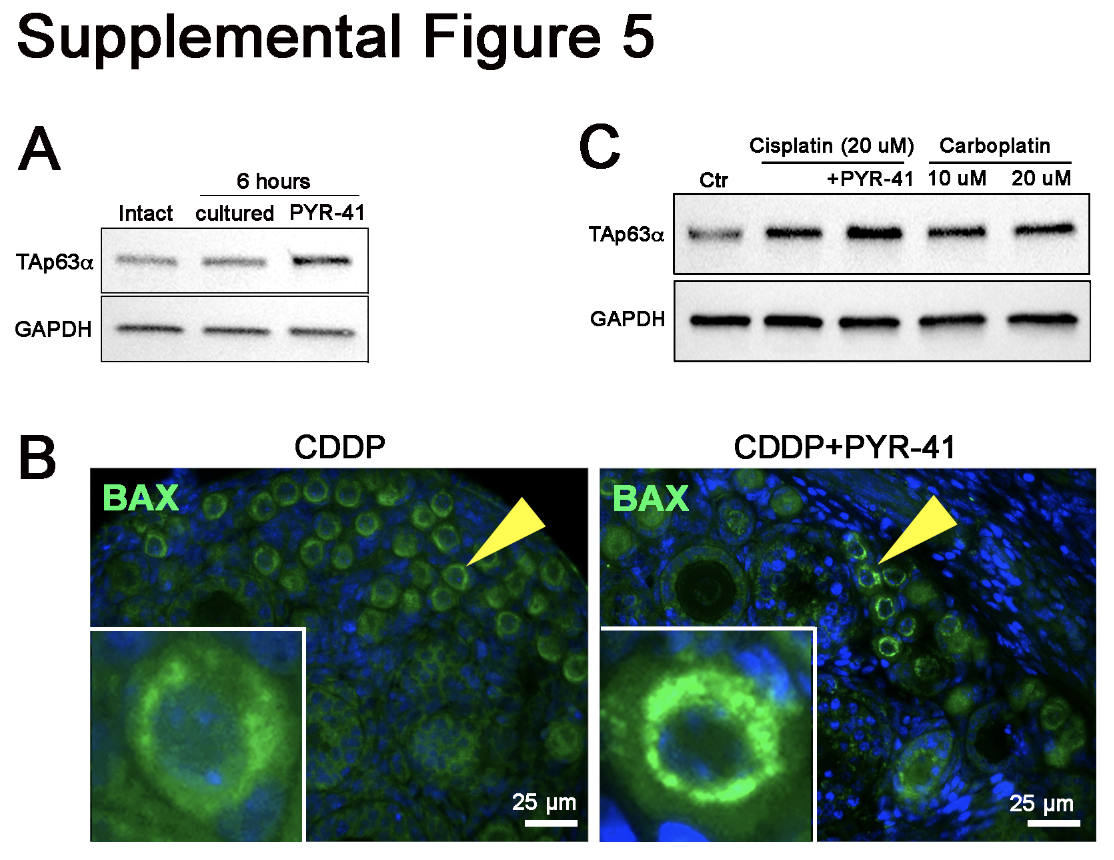
**

**Figure S5. PYR-41 contributes to the accumulation of TAp63α in oocyte death within primordial follicles. A.** Expression of TAp63α and GAPDH in ovaries cultured *ex vivo* with or without PYR-41 for 6 hours. **B.** BAX expression in oocytes of primordial follicles from ovaries treated with CDDP and CDDP+PYR-41. Expression is marked by yellow arrowheads. **C.** Immunoblot analysis of TAp63α and GAPDH in ovaries treated with solvent (Control, Ctr), CDDP, CDDP+PYR-41, or Carboplatin for 6 hours.
